# Key considerations for assessing soil ingestion exposures among agricultural workers

**DOI:** 10.1038/s41370-021-00339-z

**Published:** 2021-06-02

**Authors:** Sara N. Lupolt, Jacqueline Agnew, Thomas A. Burke, Ryan David Kennedy, Keeve E. Nachman

**Affiliations:** 1 Department of Environmental Health & Engineering, Johns Hopkins Bloomberg School of Public Health, Baltimore, MD USA; 2 Johns Hopkins Center for a Livable Future, Johns Hopkins Bloomberg School of Public Health, Baltimore, MD USA; 3 Risk Sciences and Public Policy Institute, Johns Hopkins Bloomberg School of Public Health, Baltimore, MD USA; 4 Department of Health Policy and Management, Johns Hopkins Bloomberg School of Public Health, Baltimore, MD USA; 5 Department of Health, Behavior and Society, Johns Hopkins Bloomberg School of Public Health, Baltimore, MD USA

**Keywords:** Soil ingestion, Agriculture, Exposure factor, Farmers

## Abstract

**Background:**

Soil ingestion is a critical, yet poorly characterized route of exposure to contaminants, particularly for agricultural workers who have frequent, direct contact with soil.

**Objective:**

This qualitative investigation aims to identify and characterize key considerations for translating agricultural workers’ soil ingestion experiences into recommendations to improve traditional exposure science tools for estimating soil ingestion.

**Methods:**

We conducted qualitative in-depth interviews with 16 fruit and vegetable growers in Maryland to characterize their behaviors and concerns regarding soil contact in order to characterize the nature of soil ingestion in the agricultural context.

**Results:**

We identified and discussed four emergent themes: (1) variability in growers’ descriptions of soil and dust, (2) variability in growers’ soil contact, (3) growers’ concerns regarding soil contact, (4) growers’ practices to modify soil contact. We also identified environmental and behavioral factors and six specific agricultural tasks that may impact soil ingestion rates.

**Significance:**

Our investigation fills an important gap in occupational exposure science methodology by providing four key considerations that should be integrated into indirect measurement tools for estimating soil ingestion rates in the agricultural context. Specifically, a task-based framework may provide a structure for future investigations of soil contact that may be useful in other populations.

## Introduction

Occupational health research with agricultural workers typically focuses on injuries and/or inhalation and dermal exposure to pesticides, but ingestion of soil may be an ongoing source of exposure to contaminants. Soils used for food production may be contaminated due to the natural occurrence, current and historical industrial activity (e.g., fossil fuel combustion, waste incineration), legacy uses of lead-based paint and leaded gasoline [1–5], and pesticide applications [6, 7]. Agricultural workers cultivate crops and tend livestock, though there are a variety of other tasks related to the agricultural work for which these workers are responsible, depending on the farm operation (e.g., pesticide application, grounds maintenance, equipment repair, and small construction or building projects). Agricultural workers (in both urban and rural settings) who routinely engage specifically in the cultivation of crops (hereafter termed growers) and have frequent and direct contact with these soils may be at increased risk for adverse health effects, though these risks have not been well characterized [8–10].

Exposure to contaminants in soil can occur through dermal contact with soil/dust, inhalation of soil/dust particles, and ingestion of soil/dust particles. Of these, ingestion is generally hypothesized to be the dominant route [2, 11, 12]. Because soil is usually not intentionally consumed, traditional dietary exposure assessment methodologies (e.g., 24-h dietary recalls, food frequency questionnaires) that involve participant recall are not likely to produce accurate estimates of soil ingestion. Given the incidental nature of soil and dust ingestion, investigation and quantification of time activity factors related to specific activities and occupations and non-dietary ingestion factors such as frequency of hand-to-mouth behaviors have traditionally been used for modeling and estimating rates of soil ingestion [13]. Existing research on time activity factors has focused on a very broad collection of activities for the general population, and most research on non-dietary ingestion factors has focused on children’s hand and object to mouth behaviors [14, 15], with only a few studies focused on adult non-dietary ingestion factors in limited occupational settings [16, 17]. Yet little is known about non-dietary ingestion patterns of adults, and the use of children’s patterns to estimate adult growers’ soil and dust ingestion is questionable.

While quantitative estimates of activity pattern and non-dietary ingestion factors are critical for characterizing soil ingestion exposure, several environmental, behavioral, and timing considerations may impact the frequencies and durations of these actions. Limited work has sought to characterize the specific tasks and activities that comprise agricultural work and their relative influence on soil ingestion[18]. Qualitative methods are under-utilized in exposure science research and rely on non-numeric data to understand the complex nature of individuals’ experiences and beliefs, and how they may impact actions and subsequent exposure. Here we employ in-depth interviews (IDIs) to identify and characterize key tasks and important considerations for understanding growers’ soil contact experiences to improve exposure science tools for estimation of soil ingestion in agricultural settings.

## Methods

We used purposive sampling to identify fruit and vegetable growers in the state of Maryland who would be eligible for recruitment in two ways. First, we used Maryland’s Best website, an online database of farms and farm businesses in Maryland maintained by the MD Department of Agriculture. We also referenced our team’s internal database of farmers who participated in a previous (Safe Urban Harvests) study of farmers growing in Baltimore City [19, 20]. We then recruited (i.e., reached out to and communicated with eligible farmers) by email and direct networking at conferences and local community events. Growers were eligible if they were currently a farm owner/manager, farm employee, or community gardener in Maryland; ≥18 years of age; had completed farm activities related to food production (e.g., planting, harvesting, weeding, mulching) within the past twelve months; and expected to be engaged in farm activities in the upcoming twelve months. Informed consent was obtained from all participants and all IDIs were audio-recorded. All participants completed a brief questionnaire containing basic demographic identifiers (e.g., age, gender, education, hours worked). Participants were offered a $20 gift card as compensation for their time. All study tools and protocols were reviewed and approved by the Johns Hopkins Institutional Review Board (IRB00009866).

We conducted IDIs with fruit and vegetable growers in Maryland at their farms between January and February 2020. The first author (SL) conducted and took notes during all interviews using a semi-structured discussion guide (Supplemental Materials) designed to gather information about specific agricultural tasks and site characteristics. Briefly, the guide began with questions asking growers to describe their operation, the distribution of the labor onsite, and a typical workday. Next, participants were asked to provide detailed descriptions of specific tasks/work activities (e.g., planting, irrigation, weeding, harvesting) mentioned during the interviews. The guide also included questions about soil contact (including incidental ingestion) and methods of increasing or decreasing soil contact (e.g., wearing personal protective equipment (PPE), typical work attire, and hand hygiene facilities onsite). Finally, the guide contained questions to solicit information about health and safety concerns experienced by growers while working onsite. Given the formative nature of this research, we determined saturation for specific topic areas through an iterative process of transcript review following each new IDI.

In accordance with the IDI guide, the interviewer consistently used the word “soil” in all questions posed to participants in the first few interviews. Consistent with the framework approach that includes processes for iterative review, through the close reading of the initial transcripts and reflexive journaling we noticed many growers also used the words “dirt” and “dust” in addition to “soil” and began to probe further about these words to determine whether the use of these words suggests different concepts that are meaningful to growers, and how growers’ understanding of these terms may relate to definitions commonly used by exposure scientists. Because the close reading and reflexive journaling occurred throughout the interview process, and the addition of the probe occurred after the first two interviews, it is unlikely this change differentially impacted the content of the interviews. All audio recordings were transcribed verbatim using the NVivo transcription service and verified by SL who listened to all of the recordings while reading the automatically generated transcripts to verify the transcription accuracy. One audiorecording file was lost prior to transcription and was not included in subsequent analyses.

We used an adapted framework approach for analysis, which is a method of analyzing qualitative data appropriate for answering pre-determined research questions that is also responsive to the discovery of emergent themes [21]. The first author (SL) coded each transcript using a combination of inductive coding and deductive coding methods [22]. In the first round of coding, SL developed a set of deductive codes designed to capture key concepts informed by the questions included in the IDI guide. After coding each transcript with the set of deductive codes, SL re-read all transcripts looking for emergent themes which she coded inductively. As an example, SL coded the six tasks most frequently mentioned by growers, (i.e., bed preparation, planting, pest management, harvesting, weeding, and produce handling) as emergent themes. Next SL aggregated similar codes together to compile the data across themes. Within the re-aggregated codes, SL conducted the second round of inductive coding, noting emergent themes within each set of aggregated codes. To develop the analytical frameworks, we identified key exposure science concepts a priori (e.g., definitions of soil and dust, routes of exposure, and the hierarchy of controls) and iteratively identified emergent themes from the data and then mapped the data to the emergent frameworks. The resulting emergent themes were discussed, and frameworks and figures were refined by all co-authors.

## Results

We conducted sixteen IDIs ranging from 21 to 92 min (mean = 50 min). The majority of growers interviewed were female (*n* = 9), working full time (≥35 h per week) (*n* = 10) and working in Baltimore city (*n* = 9) (i.e., hereafter described as “urban”; growers working outside of Baltimore city are described as “rural”) (Table 1). When growers had previous experiences on operations both inside (urban) and outside Baltimore city (rural), we classified them according to the location of their operation at the time of the interview.

**Table 1:** Demographic characteristics of fruit and vegetable growers interviewed (*n* = 16).

	*n*	%
Age^a^		
20–29	4	25
30–39	4	25
40–49	1	6
50–59	2	13
60+	4	25
Female	9	56
Growing within Baltimore city	9	56
Highest level of education obtained		
Associates degree	1	6
Bachelor’s degree	11	69
Professional degree beyond a bachelor’s degree	3	19
Graduate degree	1	6
Employment status		
Employed, working full-time	10	63
Employed, working part-time	1	6
Not employed, looking for employment	0	0
Not employed, NOT looking for employment	1	6
Retired	3	19
Other	1	6
Hours worked per week		
Full time (≥35 h/week)	10	63
Part time (<35 h/week)	6	38
Compensation		
Salary	3	19
Hourly wage	1	6
Not paid	6	38
Other	6	38

^a^One participant declined age.

We identified and discussed four emergent themes: (1) variability in growers’ descriptions of soil and dust, (2) variability in growers’ soil contact (3) growers’ concerns regarding soil contact (4) growers’ practices to modify soil contact. These themes suggest several key considerations for designing tools for estimating soil ingestion in the agricultural context.

### Variability in growers’ descriptions of soil and dust

**Fig. 1:**
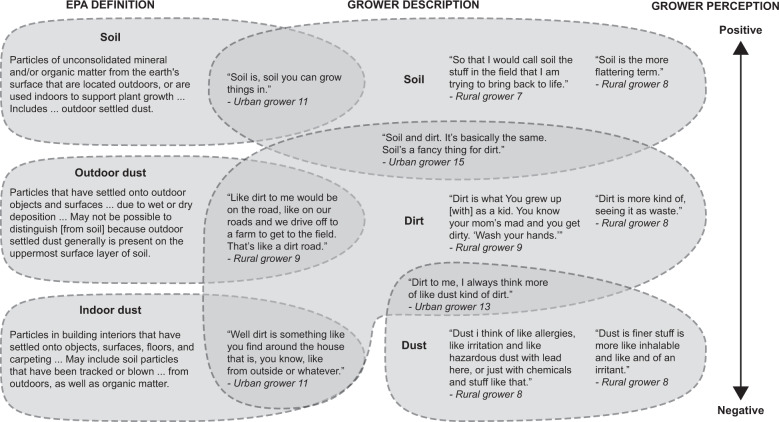
Comparison of demonstrative quotations of growers’ descriptions of soil, dirt, and dust classified according to EPA definitions of soil and dust and growers’ perceptions. Growers’ perception scale is located on right of figure with positive perceptions at the top, and negative perceptions at the bottom.

The US EPA Exposure Factors Handbook (EFH) defines soil, outdoor settled dust, and indoor settled dust separately [23] (Fig. 1). For the purposes of soil and dust ingestion estimation, EPA considers soil ingestion the ingestion of both soil and outdoor settled dust. Dust ingestion involves the ingestion of indoor settled dust only. Of note, the EPA EFH does not use nor explicitly define the term “dirt” or explain how it relates to the terms, soil, outdoor settled dust, or indoor settled dust, though a separate body of research has investigated the relationship between soil and indoor dust [24, 25].

Although growers were not asked specifically to define each of these terms, their explanations of why and how they use each of these terms may pose translational challenges between growers and exposure scientists when characterizing soil exposure in an agricultural context. Most growers used the words soil, dust, and dirt interchangeably when describing their soil contact experiences (Fig. 1). Though we asked growers about “soil” contact while utilizing the EPA EFH definition, it is unclear whether all growers were consistently including only exposure to soil and outdoor settled dust (but not indoor dust) in their responses. This emergent theme highlights the translational challenges between growers and exposure scientists’ definitions of soil and dust as well as growers’ frequent use of the non-specific word dirt. For example, when a grower used the word dirt in her explanation of soil contact, it was difficult to classify her experience within the EPA EFH definitions. When asked directly, one grower did not see or acknowledge any distinction between the terms soil, dirt, and dust. Other growers’ descriptions of dirt drew on aspects of both EPA’s definitions of soil and dust. Several growers described dirt as being more closely related to dust than soil. Others described dirt as more related to soil than dust. One grower described dirt as more similar to EPA’s definition of indoor dust:

*“Dirt to me, I always think more of like dust kind of dirt. […] I think, oh, I’m gonna clean my house.”* *(Urban grower 13)*

A different grower’s description of dirt was more similar to EPA’s definition of outdoor settled dust than indoor settled dust or soil:

*“I would say dirt. It is different, […] like dirt to me would be on the road, like on our roads and we drive off to a farm to get to the field. That’s like a dirt road. The soil is the living part that we want to keep alive and maintain and treat with respect. So that I would call soil the stuff in the field that I am trying to bring back to life and dirt I would call, you know, the place where weeds don’t grow.”* *(Rural grower 7)*

This finding raises important concerns about the accuracy of data collected and highlights the risk of exposure misclassification in soil exposure assessments.

A related theme that emerged and may help exposure scientists better classify growers’ descriptions of soil contact, is whether the term used evoked positive or negative connotations. Specifically, we observed a continuum in which growers’ descriptions of soil contact had positive connotations, while descriptions of dirt and dust contact were more likely to have negative connotations (Fig. 1). Even among growers who used the words soil and dirt interchangeably, they acknowledged that soil generally evoked more positive connotations than dirt.

*“Soil and dirt. It’s basically the same. Soil dirt, dirt soil. Soil’s a fancy thing for dirt. A sanitation engineer, a trash man, you know.”* *(Urban grower 15)*

Specifically, a consensus emerged that the positive connotations of soil were associated with feelings and images of life, whereas dirt evoked negative connotations associated with a lack of life. One grower argued that using the term soil has an implicit notion of both life and care:

*“I see soil as something that is enriched. It’s living. It has some life in it. It’s being managed and being tended to. Versus dirt being you know, a pile of top that might be just used for capping a brownfield site. […] it obviously is soil that got scraped off of the yard, but it’s been sitting somewhere and is just being used to like hold a place. Where soil is something that you have to manage, you want to keep it covered, you want to take care of it.”* *(Urban grower 16)*

Another grower, while not specifying indoor or outdoor, emphasized dust has a more negative connotation than dirt:

*“Yeah I think dust is finer stuff, is more like […] an irritant […] than dirt is. Dirt like, I think it’s like dirty. But like dust, I think of like allergies, like irritation, and like hazardous dust, like dust with the lead there, or dust with chemicals and stuff like that.”* *(Rural grower 8)*

Growers’ use and perceptions of terms used to describe soil and related media is an important consideration for future investigations of soil contact in agricultural contexts.

### Variability of growers’ soil contact

Although understanding soil ingestion was the motivation of this research, our conversations demonstrate that ingestion is part of a multi-route, multi-pathway exposure scenario, and inquiring directly and specifically about soil ingestion likely underestimates exposure in the agricultural context. Previous observational studies on unintentional ingestion among industrial [26] and agricultural workers [18] suggest that consideration of PPE use and work attire may influence the nature and frequency of hand and object-to-mouth behaviors, which often result in transfer of soil into the mouth.

We asked growers to describe a typical workday and the tasks or activities that result in the most soil contact. Overall, growers described six distinct, routine tasks they complete while growing edible crops (i.e., bed preparation, planting, pest management, irrigation, harvesting and produce handling). Among urban growers, all but one indicated that planting was the most soil contact intensive task. The urban grower who did not mention planting mentioned bed preparation. Among rural growers, bed preparation and pest management/weeding were tasks most frequently mentioned as resulting in the greatest soil contact. While a particular task may directly impact rates of soil ingestion, a broader host of environmental and behavioral factors may also contribute to soil ingestion within a particular task.

**Fig. 2:**
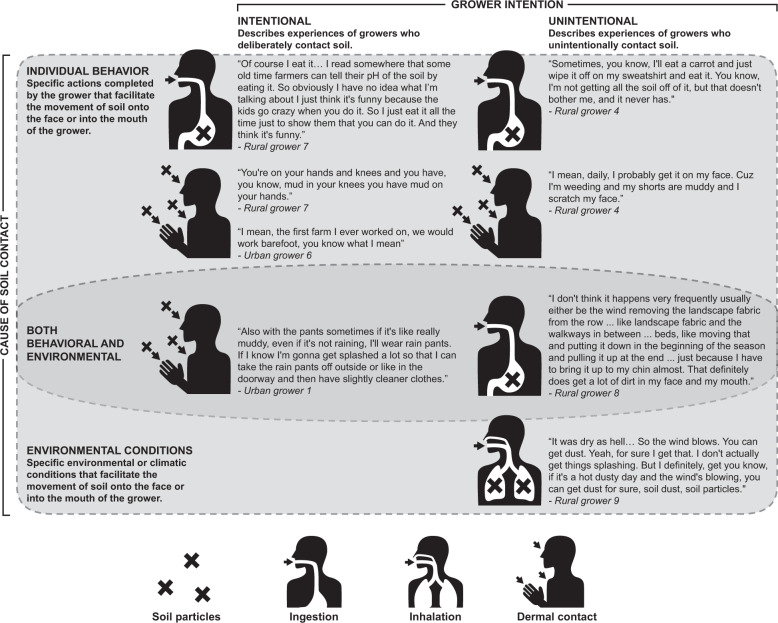
Demonstrative quotations from grower interviews classified according to two sets of emergent factors: grower intention (unintentional or intentional) and cause of soil contact (individual behavior and environmental conditions). Image to the left of quotation indicates route of exposure illustrated by quotation.

When asked more generally about soil contact, including getting soil on the face or in the mouth, most growers provided vivid descriptions of a variety of soil contact experiences occurring via other (i.e., inhalation and dermal) routes. Using growers’ descriptions of soil contact, we developed a framework (Fig. 2) to classify soil ingestion experiences based on two emergent factors: worker intention (unintentional or intentional) and cause of the soil contact event (environmental or behavioral). Individual behaviors may be intentional in which the grower knowingly and deliberately makes contact with soil or unintentional, in which the grower knowingly, but not deliberately makes contact with soil. No growers described pica behavior, and only one grower reported intentionally tasting soil on occasion. Growers’ descriptions of both intentional and unintentional soil contact events generally included behavioral factors (e.g., specific actions completed, or decisions made by the grower that facilitate the movement of the soil to the face or mouth of the grower) as a cause of the soil contact event. As an example, walking barefoot is a behavior that increases dermal contact with soil and may contribute to ingestion through the take-home pathways. Growers track soil into their homes where it may settle and later be incidentally inhaled as indoor dust. Furthermore, behavioral factors that at any time increase the frequency or duration of hand to mouth or object to mouth contact events in the presence of soil may contribute to soil ingestion.

Two growers mentioned both behavioral factors and environmental factors (e.g., specific environmental or climatic conditions) that facilitate the movement of the soil to the face or into the mouth of the grower. After classifying each soil contact event within this framework, we matched each description according to the route of exposure it described (Fig. 2). Both grower intention and perceived cause of soil contact are important for understanding the context in which soil contact events occur in agricultural settings. Our findings suggest that assessments of soil ingestion should include direct inquiries regarding other routes of exposure, which may occur simultaneously and differentially influence rates of soil ingestion. As an example, all instances of soil contact occurring through inhalation included only environmental factors as the cause of soil contact.

### Growers’ concerns regarding soil contact

Although growers described a wide range of soil contact activities, they described exposure to soil as ubiquitous, unavoidable, and not necessarily a predominant concern. This finding demonstrates a gap between the salience of soil contact between growers and exposure scientists and suggests future tools for estimating soil ingestion should prioritize querying growers on contextual factors such as growing practices and behaviors rather than soil ingestion directly.

We asked growers to describe health and safety concerns they have while working and then classified their concerns according to five types of occupational hazards (e.g., chemical, biological, physical, safety, and psychosocial) commonly present in the workplace (Table 2). Of note, seven growers initially answered that they had no concerns related to their work, though as each conversation continued, all but two growers ultimately mentioned at least one occupational hazard. Chemical and safety hazards were the most frequently mentioned. Chemical hazards ranged from metals (lead was the only metal specifically mentioned) in the soil to diesel exhaust from nearby traffic and/or machinery used on site, and the use of chemical pesticides and corrosive agents for cleaning machinery. Two urban growers raised concerns regarding exposures to soil contaminants for specific vulnerable populations (e.g., pregnant women and children). Safety hazards ranged from risk factors for traumatic injury associated with overwork and/or misuse of tools or while conducting unfamiliar tasks as well as concerns about nearby traffic. Four urban growers raised concerns regarding personal safety from violence, a psychosocial risk factor. Three urban growers raised concerns about potential biological hazards from discarded needles found in soil. Other specific biological hazards included contact with pathogens (e.g., *Staphylococcus*) and animal feces. Two growers mentioned concerns regarding skin reactions—either from poison ivy or from exposure to resins found in plant leaves, but not from soil. The only physical hazard mentioned by one rural grower was heat stress. Other physical hazards such as sun exposure or exposure to cold were not mentioned by any growers (Table 2). One rural grower mentioned broader ecological concerns regarding erosion and soil tillage practices, though the link to the grower’s health and safety was not elucidated.

**Table 2:** Summary of workplace hazards discussed by growers.

Chemical	
Soil contamination (e.g., lead, pesticides)^a^	
Application of pesticides, fertilizer, diatomaceous earth	
Use of corrosive chemical cleaning agents	
Inhalation of diesel fumes (from farm equipment and traffic nearby)	
Asbestos present in nearby buildings	

Biological	
Skin irritation from plants (e.g., celery and poison ivy)	
*Staphylococcus* infections^a^	
Mouse feces	
Used needles^a^	

Physical	
Heat stress	

Safety	
Falls from ladders	
Used needles	
Risk of getting hit because farm is located near busy road	
Injury from incorrect use or accidents with power tools, tractor, knives	

Psychosocial	
Working alone (e.g., is there someone around who could help me?)	
Fear of violence because farm is located in certain neighborhood	
Fear of vandalism and/or personal safety (e.g., no fences; site is not well-lit at night)	

^a^Indicates concerns related to soil contact.

Although exposure to chemical contaminants in soil was not the most frequently mentioned hazard, we asked growers to describe their familiarity with soil testing practices. Many growers had their soil tested, though the specific type of test and the analytes included in the test were not always clear. Most rural growers described nutrient testing in detail but had less experience with contaminant testing. Urban growers expressed more familiarity with heavy metal contaminant testing; two growers even commented how to interpret contaminant test results (e.g., what concentration of lead would be acceptable in regard to different state and federal standards). No growers had experience testing soils for pesticides, polycyclic aromatic hydrocarbons, or other non-metal soil contaminants, and one grower described the perceived futility of doing so:

*“Maybe some persistent pesticides. But even if there had been some presence of glyphosates [sic], I would just continue to farm anyway because there would have been no way for us to get away from it.”* *(Urban grower 16)*

A few growers had previous farming experience in both urban and rural contexts and described how soil contamination concerns vary between urban and rural contexts. A rural grower suggested that metal contaminants were more likely concerns in urban areas:

*“And this is probably a little prejudice on my part, but I would think that the urban farms would have more concern about the heavy metals than maybe the farms up here. But that could just be my ignorance of not knowing what other people are adding to their soils or where the heavy metals could come from.”* *(Rural grower 4)*

Though one urban grower’s experiences directly contradicted this suggestion:

*“I guess my perspective also is like having also lived in very rural areas […] where I saw people’s dump piles, you know, in the woods […] And it’s very picturesque […] it’s very pastoral. But like that’s somebody’s old oil drum right there. […] my perspective is there’s contamination everywhere. And it’s not necessarily just an urban issue. I think part of that is a social stereotype. […] I think there are a lot of sources that people discount in rural areas.”* *(Urban grower 1)*

Our conversations demonstrate that even though soil contact was not a salient concern, growers were generally aware of the issue of soil contamination, and in many cases, were even familiar with testing practices to address potential soil contaminant concerns. This finding suggests framing future investigations to estimate soil ingestion around specific activities (i.e., farming related tasks) and related behaviors (i.e., personal preferences and ways of completing activities) rather than a health or safety concern may provide more contextual information to better estimate soil ingestion.

### Growers’ practices to modify soil contact

Although exposure to soil contaminants was not a dominant concern among growers, our conversations revealed that many took intentional steps to modify (i.e., increase or reduce) their contact with soil. Understanding current practices for modifying soil contact may help occupational and public health practitioners develop more actionable and appropriate guidance for growers to reduce soil contact and improve estimates of soil ingestion.

Most growers discussed routine and conscious behaviors to modify soil contact. One grower mentioned intentionally increasing soil contact, unrelated to a specific activity.

*“I will occasionally go barefoot when I really feel like I need to be grounded some more.”* *(Rural grower 2)*

At least 5 growers (3 urban and 2 rural) indicated they do not ever take any actions to reduce soil contact, though most described a least one action to reduce soil contact.

We classified growers’ descriptions of soil contact reduction behaviors according to the Hierarchy of Controls as described by the National Institute for Occupational Safety and Health (NIOSH) [27]. This classification tool is widely used to classify and prioritize occupational interventions for reducing exposures in workplaces. According to the hierarchy, types of controls are ranked according to their level of feasibility and effectiveness in the following order: elimination of the hazard (most effective), substitution, engineering, administrative, and PPE (most feasible).

Among growers in this study, only engineering, administrative, and PPE controls were cited. No growers described substitution or elimination controls for reducing soil contact, which could include growing in soil-less systems. Three growers described engineering controls such as mulching paths or installing landscaping tarps on or between growing beds to reduce potential soil splash during rain and irrigation events and the prevention of dust during drier periods. One grower described using height adjustment while working to bring the work to a more appropriate ergonomic level. Several also described no-till cover cropping techniques to improve soil health and also reduce the risks of erosion. The most common ways growers described reducing soil contact were through administrative controls (e.g., policies or requirements for hand hygiene) and PPE (e.g., wearing gloves or specific clothing attire).

No grower mentioned administrative controls, including policies, requirements, or training specific to reducing soil contact. One grower attributed lack of training received specifically to farm size:

*“I think every farm I’ve worked at was too small to sort of have the system.”* *(Urban grower 10)*

Some growers mentioned familiarity with or having received information materials on Good Agricultural Practices or Good Handling Practices from their local USDA Extension office. Of note, the emphasis of this audit-based program is to minimize microbial food safety risks during production, harvesting, handling, and storage of fruits and vegetables, so its relevance as an occupational health program for reducing soil contact is unclear. One grower had taken a private pesticide applicator certification course. Most commonly, growers described on the job training either from experience:

*“I’ve just been doing it and learning it as I go”* *(Urban grower 6)*

Or from peer-to-peer or manager to employee instruction:

*“I train them on just using hand tools properly. Nobody drives the tractor unless they’re trained.”* *(Rural grower 7)*

No grower mentioned formal policies or requirements on site for handwashing but emphasized that it was highly encouraged and occurred often, though the extent to which frequent handwashing is possible may be dependent on the restroom and break facilities at each site. Four rural growers and one urban grower lived on site and thus had restroom and kitchen facilities in their homes. Two sites had a toilet facility onsite in buildings associated with the farm. Two sites had composting toilets, and another had a portable toilet. One urban grower, who also works as a landscaper, mentioned that not all worksites have restroom access. Four sites had no associated restroom facility and two sites routinely relied on restrooms in a nearby community center. Most growers described a designated place to stop to eat lunch or rest, typically at a picnic table outdoors and in the shade. Growers who live on site described routinely returning to their homes for meals. Three growers mentioned distinct, enclosed shelters for rest and food consumption. Even though running water was available at all sites, distinct handwashing facilities (including soap) were not always available at all sites.

All growers mentioned using PPE to reduce soil contact, either as part of their typical work attire or additional equipment worn while engaging in specific tasks. A typical outfit generally consisted of long pants, long or short sleeves, and closed-toed shoes (i.e., either sneakers or boots). One grower mentioned an employee who often works barefoot. Temperature and moisture conditions were two emergent themes that impacted what clothing growers may wear.

*“It depends on the weather […] There’s really no consideration other than, like, how I can stay comfortable while working as far as temperature goes.”* *(Urban grower 10)*

*“It’s just easier when you’re not expecting rain.”* *(Urban grower 1)*

Gloves were the most common form of attire worn specifically for worker protection. The types of gloves growers use vary from reusable gardening gloves to one-time use disposable nitrile or latex gloves and were constructed of a variety of materials including cotton cloth, nylon, plastic, nitrile or latex. Several growers described using gloves with a rubber-like coating, though they were not certain of the material. The interviewer probed growers during which tasks they would typically wear gloves and why. The most commonly reported reason for wearing gloves was to protect the hands generally, both to prevent direct injury and the development of callouses.

*“When you use the buckets to water or take the buckets or you picking up something that you use when you dig your gloves for that, too, because it can be hard on your hands.”* *(Urban grower 15)*

One grower emphasized the importance of wearing gloves for consumer protection, rather than worker protection.

*“We do sometimes wear gloves when we’re harvesting produce. That’s more like protection of the people who are consuming our produce.”* *(Rural grower 8)*

Of note, one grower explicitly clarified that protection from soil was not a primary reason for wearing gloves:

*“The only time I would ever, like use a pair of gloves is if I was going to be doing some task with my hands, if it was gonna be a long, drawn task. But not to protect myself against the soil.”* *(Urban grower 16)*

Growers indicated a wide range of frequency with which they wear gloves. Responses ranged from constantly:

*“I wear them 24 [/7]. Haha, no, I don’t sleep in them, right? I wear them all the time”* *(Rural grower 9)*

to rarely:

*“I mean, once in a while put gloves on.”* *(Rural grower 4)*

Three growers adamantly stated they never wear gloves, though most stated that there are only some specific tasks for which they do wear gloves. The most common reason for not wearing gloves was to retain manual dexterity. Seeding was one task two growers specifically indicated they would not wear gloves:

*“But a lot of my job is tactile, and I can feel the difference between a weed stem and a lettuce stem, and I need to feel it […] I won’t seed with them on because I can’t feel the seeds.”* *(Rural grower 4)*

Masks were another PPE item mentioned, though less frequently than gloves. The tasks associated with wearing masks were related to pesticide application, animal care, and/or the use of mechanized farm equipment that emitted diesel. Of note, all interviews were conducted between January and February 2020, at the beginning of the COVID-19 pandemic when face mask demand was high and prioritized for health care workers. One grower indicated difficulty obtaining masks at this time:

*“I actually tried to order some last night- More N95 masks- and everybody’s sold out… Everybody’s sold out. Like. I can’t get a mask. I mean, I had some this winter, but you start, and you order new stuff with the spring and I can’t find em.”* *(Rural grower 14)*

In addition to asking growers about typical work attire, the interviewer also probed to assess activities and behaviors that may impact the take-home pathway route of exposure (e.g., transporting soil and/or chemicals from the farm back to or into the home). No growers indicated they changed out of work clothes onsite before traveling home. However, changing footwear before leaving the site was common:

*“I usually change my boots. When I first started here, I just sort of wore everything everywhere. That was not so good for my car, for my house. So I got like boots that I try to keep here. I wear sneakers to my car, and I drive back home with sneakers. Sometimes I like forget about something. And I walk around the farm for a second in my sneakers because I’m like, oh, I got to harvest a bunch of kale for myself or I forgot to turn off the water or whatever.”* *(Rural grower 8)*

All growers described laundering their work clothes in a washing machine at their primary residence. Most growers described washing all their work clothes together, though often separately from the rest of their household’s laundry.

*“They get separated from my wife’s stuff because years ago I learned not to mix stuff. The typical guy thing, you know, throw it all in the same load. It doesn’t work like that.”* *(Rural grower 9)*

Our conversations with growers revealed limited use of elimination, substitution, engineering, and administrative controls to reduce soil contact among growers and frequent, though task and situation dependent, use of PPE controls. This finding reiterates the value of the hierarchy of controls framework for prioritizing more effective controls (e.g., engineering controls) whenever feasible. Given the variability in the type and context of PPE use, our findings suggest further investigation of the frequency, context, and rationale of these practices should be incorporated into future tools and estimations of soil ingestion in the agriculture context.

## Discussion

We interviewed 16 farmers growing fruits and vegetables in urban and rural contexts in Maryland to characterize how, when, and in what context they may be exposed to and incidentally ingest soil. To our knowledge, this study is the first to investigate soil ingestion and contact among agricultural workers for the purpose of improving soil exposure assessment methods. The qualitative nature of this study provides important context for the activity, environmental and behavioral factors that may impact rates of soil ingestion among fruit and vegetable growers, a highly exposed occupational population.

Our conversations revealed variability in growers’ descriptions of soil and dust and their soil contact experiences, which raises two key methodological considerations for exposure scientists using text-based tools (e.g., surveys or interviews) for indirect measurement of soil exposure: (1) the differential use of foundational terms (e.g., soil and dust) between exposure scientists and study participants may pose translational challenges that undermine the accuracy of existing tools and (2) a framing or emphasis on soil ingestion exclusively neglects the multiple routes and pathways of soil exposure that may simultaneously contribute to and modify soil ingestion rates in the agricultural context. This omission may result in significant misclassification (and likely underestimation) of exposure. In addition, we identified several activity-related (e.g., tasks), environmental (e.g., temperature and moisture conditions), and behavioral (e.g., use of PPE) factors which may modify rates of soil ingestion in the agricultural context. We also acknowledge that seasonal variability related to activity, environmental, and behavioral factors may further modify rates of soil ingestion both within and across seasons. Given that growers described variability in soil contact while engaging in specific tasks, future research should explore the frequency and duration of these tasks to better quantify the time-activity patterns of agricultural workers. Additional qualitative investigations of the nature and context in which each task is completed may elucidate the relative influence and magnitude of activity, environmental and behavioral factors on soil contact.

Farm size was another factor hypothesized by growers to impact the nature of administrative controls for reducing soil contact. Because this was an emergent theme, we were unable to compare growers’ experiences working on farms of different sizes, and a potential limitation of our study is the homogeneity of growers interviewed. All study participants worked on small, independently owned farms in Maryland. While these farm types are commonly found in the US, they do not necessarily represent most or all farm operations; however, what we learned about soil contact on smaller farms is likely relevant to other operations, though additional investigation is needed.

Recommended soil ingestion rates published in the EPA EFH are frequently used to inform risk assessments for contaminated lands and derive public and occupational health guidance values for contaminants in soil, though the agency’s assessment of quality and confidence in these estimates is low. Given known methodological limitations associated with direct measurement tracer methods used to estimate soil ingestion rates [28], exposure scientists rely on a broader range of indirect measurement approaches to estimate rates of soil ingestion. Incorporating the key findings of this study could result in critical improvements to existing indirect measurement tools (surveys and direct and videographic observations) that greatly improve the accuracy and utility of data collected for soil ingestion estimation both for the general population, and for agricultural workers.

For example, emerging evidence suggests agricultural soils may be reservoirs for persistent pesticides [29] which may pose ongoing and additional risks to agricultural workers, though this pathway is rarely considered in epidemiological studies and regulatory practice. Regulatory risk assessments conducted in support of pesticide registration at the US EPA do not consider exposures occurring via soil ingestion. More robust characterization of both soil exposure and estimates of soil ingestion in the agricultural context could generate the evidence needed to shift regulatory decisionmaking for pesticide regulation, improve occupational health guidelines, and reduce adverse health outcomes among agricultural workers. Similarly, integration of soil exposure in epidemiological studies of agricultural workers’ exposures to pesticides may reduce previously unaddressed exposure misclassification.

Soil ingestion is likely a key pathway of exposure to contaminants for agricultural workers, but methodological limitations and poor characterization of the context in which soil ingestion occurs have prevented robust estimates of soil ingestion for this population to date. Our study identified key methodological and context-specific considerations useful in improving existing indirect measurement tools (e.g., surveys and/or direct observation.) Future research should apply these key considerations to quantify the extent to which the activity, environmental and behavioral factors we identified may impact the frequency and duration of key non-dietary ingestion factors (i.e., hand and object to-mouth behaviors) and act as effect modifiers. For example, future surveys should recognize the variability in growers’ descriptions of soil and dust and instead incorporate definitions (whether EPA given or study-specific) directly into indirect exposure assessment tools to acknowledge and minimize any disconnect in understanding between researchers and study participants and among study participants. In addition, the direct observation of growers informed by the broader spectrum of factors outlined above can be used to derive more robust estimates of soil ingestion for agricultural workers.

## Supplementary information

Supplementary Material

## References

[1] Mielke HW, Anderson JC, Berry KJ, Mielke PW, Chaney RL, Leech M (1983). Lead concentrations in inner-city soils as a factor in the child lead problem. Am J Public Health.

[2] Mielke HW, Reagan PL (1998). Soil is an important pathway of human lead exposure. Environ Health Perspect.

[3] Mielke HW, Laidlaw MA, Gonzales CR (2011). Estimation of leaded (Pb) gasoline’s continuing material and health impacts on 90 US urbanized areas. Environ Int.

[4] Schwarz K, Pickett ST, Lathrop RG, Weathers KC, Pouyat RV, Cadenasso ML (2012). The effects of the urban built environment on the spatial distribution of lead in residential soils. Environ Pollut.

[5] Yesilonis ID, Pouyat RV, Neerchal NK (2008). Spatial distribution of metals in soils in Baltimore, Maryland: role of native parent material, proximity to major roads, housing age and screening guidelines. Environ Pollut.

[6] Hood E (2006). The apple bites back: claiming old orchards for residential development. Environ Health Perspect.

[7] McBride MB, Shayler HA, Russell-Anelli JM, Spliethoff HM, Marquez-Bravo LG (2015). Arsenic and lead uptake by vegetable crops grown on an old orchard site amended with compost. Water Air Soil Pollut.

[8] Brown SL, Chaney RL, Hettiarachchi GM (2016). Lead in urban soils: a real or perceived concern for urban agriculture?. J Environ Qual.

[9] Kessler R (2013). Urban gardening: managing the risks of contaminated soil. Environ Health Perspect.

[10] Kim BF, Poulsen MN, Margulies JD, Dix KL, Palmer AM, Nachman KE (2014). Urban community gardeners’ knowledge and perceptions of soil contaminant risks. PLoS ONE.

[11] Calabrese EJ, Stanek EJ, James RC, Roberts SM (1997). Soil ingestion: a concern for acute toxicity in children. Environ Health Perspect.

[12] LaGoy PK (1987). Estimated soil ingestion rates for use in risk assessment. Risk Anal.

[13] US EPA. Exposure Factors Handbook 2011 Edition (Final Report). Washington, DC: U.S. Environmental Protection Agency; 2011. Report No.: EPA/600/R-09/052F.

[14] Xue J, Zartarian V, Moya J, Freeman N, Beamer P, Black K (2007). A meta-analysis of children’s hand-to-mouth frequency data for estimating nondietary ingestion exposure. Risk Anal.

[15] Xue J, Zartarian V, Tulve N, Moya J, Freeman N, Auyeung W (2010). A meta-analysis of children’s object-to-mouth frequency data for estimating non-dietary ingestion exposure. J Expo Sci Environ Epidemiol.

[16] Wong EY, Shirai JH, Garlock TJ, Kissel JC (2000). Survey of selected activities relevant to exposures to soils. Bull Environ Contam Toxicol.

[17] Beamer PI, Plotkin KR, Gerba CP, Sifuentes LY, Koenig DW, Reynolds KA (2015). Modeling of human viruses on hands and risk of infection in an office workplace using micro-activity data. J Occup Environ Hyg.

[18] Antwi-Agyei P, Biran A, Peasey A, Bruce J, Ensink J (2016). A faecal exposure assessment of farm workers in Accra, Ghana: a cross sectional study. BMC Public Health.

[19] Johns Hopkins Center for a Livable Future. The Safe Urban Harvests Study 2018. https://www.jhsph.edu/research/centers-and-institutes/johns-hopkins-center-for-a-livable-future/projects/urban-agriculture/safe-urban-harvests-study.html.

[20] Maryland Department of Agriculture. Maryland’s best agriculture: linking Maryland farmers with consumers. marylandsbest.maryland.gov/.

[21] Gale NK, Heath G, Cameron E, Rashid S, Redwood S (2013). Using the framework method for the analysis of qualitative data in multi-disciplinary health research. BMC Med Res Methodol.

[22] Saldaña J. The coding manual for qualitative researchers. 3rd ed. London: Sage Publications; 2016.

[23] U.S. EPA. Exposure factors handbook chapter 5 (update): soil and dust ingestion. Washington, DC: U.S. EPA Office of Research and Development; 2017.

[24] Layton DW, Beamer PI (2009). Migration of contaminated soil and airborne particulates to indoor dust. Environ Sci Technol.

[25] Calabrese EJ, Stanek EJ (2008). What proportion of household dust is derived from outdoor soil?. J Soil Contamination.

[26] Ng MG, Davis A, van Tongeren M, Cowie H, Semple S (2016). Inadvertent ingestion exposure: hand- and object-to-mouth behavior among workers. J Expo Sci Environ Epidemiol.

[27] Hierarchy of Controls Centers for Disease Control and Prevention National Institute for Occupational Safety and Health. https://www.cdc.gov/niosh/topics/hierarchy/default.html.

[28] Binder S, Sokal D, Maughan D (1986). Estimating soil ingestion: the use of tracer elements in estimating the amount of soil ingested by young children. Arch Environ Health.

[29] Bhandari G, Atreya K, Scheepers PTJ, Geissen V (2020). Concentration and distribution of pesticide residues in soil: non-dietary human health risk assessment. Chemosphere..

