# Supplementary material for: Key considerations for assessing soil ingestion exposures among agricultural workers
Source: J Expo Sci Environ Epidemiol. 2021 Jun 2;32(3):481–92. doi: 10.1038/s41370-021-00339-z (PMC8170865; doi:10.1038/s41370-021-00339-z)
Supplement: Supplementary file 1 — Supplementary Material [file 41370_2021_339_MOESM1_ESM.docx]

**Supplementary Material. In-depth Interview Guide**

| **Introductory questions**   1. Tell me a little bit about your farm or garden and the items you grow here.  - How large is the site? - How many employees/staff regularly work at the site?  1. Tell me a little bit how you started working at your farm/garden.  - How and when did you start working here? - Do you have an official job title? - How are you paid for your work?  1. Tell me about a typical workday for you on your farm/garden. |
| --- |
| **Identifying work activities**   1. What are the tasks and activities involved in growing food at your farm/garden?  - How do you prepare the farm/garden every year? How do you maintain the farm/garden throughout the season? How do you prepare the farm/garden for the off-season? - Are there any tasks you do every day you are on site? - Are there any tasks you only once or twice a season? - Does anyone at the site apply pesticides? if so, which ones, when and how?   - Do you apply pesticides? If so, which ones, when and how? - Does anyone at the site apply fertilizers? if so, when and how?   - Do you apply fertilizers? If so, when and how? |
| **Division/distribution of labor**   1. On a typical day, how do you know what tasks to do?  - Is there a farm manager who assigns tasks and/or sets a work schedule? - When you arrive on site, is there a list of tasks for you to complete? - If so, do you complete all of the tasks assigned?  1. On a typical day, how many people are working at your farm/garden?  - Does the farm owner work onsite? - Are there paid employees? - Are there volunteers?  1. How does your farm/garden divide tasks and responsibilities among workers (and volunteers?) on site? |
| **Understanding specific work activities**   1. Let's talk about: "X". (repeat for up to 5 tasks identified in question 3, as time allows)  - Can you explain to me how you do this? |
| **Understanding soil contact**   1. Of all the tasks we discussed which one(s) result in the most dirt or soil on your clothes or hands/involve the most soil contact?  - Do you recall ever getting soil in your mouth or on your face while working?* - I/You have used the word dirt and soil interchangeably. Is there a difference to you between soil and dirt? Soil and dust?, etc.*  1. Do you ever take any actions to reduce or increase your contact with soil while at work?  - How often do [specific action]? - Why do you do [specific action]?  1. Do you ever wear any personal protective equipment (e.g., gloves, masks) while working on site?  - Do you ever wear gloves while working onsite? - What kind of gloves do you wear? - When or how often do wear gloves? - Why do you (not) wear gloves?  1. Does your farm/garden have any policies, protocols or guidance about soil contact?  - Does your farm/garden provide any clothing for you to wear while working onsite? Does guidelines for work attire?   - If so, do you wear the clothing, or follow the guidelines? Why or why not? - Does your farm/garden provide gloves or any other tools to reduce soil contact?   - If so, do you use them? Why or why not? - Does your farm/garden have a designated location to eat lunch or rest?   - If so, do you eat/rest there? Why or why not? Where do you eat lunch? - Does your farm/garden have restroom and a place to clean your hands on-site?   - Do the cleaning facilities provide soap and water and/or hand sanitizers?   - Do you use the hand cleaning facilities? Why or why not? How often?  1. At the end of the day, what do you do with your work clothes?  - Do you change out of your clothes before leaving the site? - How do you wash your work clothes? |
| **Safety concerns**   1. Do you know if the soil at your farm/garden been tested for any soil contaminants (e.g., heavy metals, pesticides)? 2. Do you have any concerns about your safety or health while working on your farm/garden?  - If so, what is your greatest concern?  1. Where do you get information about safety on site?  - Where do you get information on safe use and handling of pesticides? - Have you ever received any training about your safety while onsite? |
| **Conclusion**   1. Of all the things we discussed today, which is the most important to you? 2. Is there anything you wanted to say, but didn't get the opportunity to? |

* Indicates a probe added iteratively per interviewer after initial interviews.
